# The association of dietary spermidine with all-cause mortality and CVD mortality: The U.S. National Health and Nutrition Examination Survey, 2003 to 2014

**DOI:** 10.3389/fpubh.2022.949170

**Published:** 2022-09-28

**Authors:** Huanyu Wu, Jianing Wang, Hongyan Jiang, Xin Liu, Xinyi Sun, Yunyan Chen, Cong Hu, Zheng Wang, Tianshu Han, Changhao Sun, Wei Wei, Wenbo Jiang

**Affiliations:** ^1^National Key Discipline, Department of Nutrition and Food Hygiene, School of Public Health, Harbin Medical University, Harbin, China; ^2^Department of Cerebrovascular Disease, The Fifth Afliated Hospital, Sun Yat-sen University, Zhuhai, China

**Keywords:** SPD, CVD mortality, all-cause mortality, NHANES, autophagy

## Abstract

**Background:**

Current studies on the protective effects of dietary spermidine (SPD) on cardiovascular disease (CVD) are mainly limited to animal studies, and the relationship between dietary SPD and CVD mortality remains inconclusive.

**Objective:**

This study aims to evaluate the association between dietary SPD intake and CVD and all-cause mortality.

**Methods:**

A total of 23,894 people enrolled in the National Health and Nutrition Examination Survey (NHANES) from 2003 to 2014 were recruited for this study. The dietary intake of SPD from 11 specific food origins and total SPD was categorized into tertiles or quartiles. Cox proportional hazard regression models were developed to evaluate the association of SPD intake with CVD and all-cause mortalities.

**Results:**

Among the 23,894 participants, 2,365 deaths, including 736 deaths due to CVD, were documented. After adjustment for potential confounders, compared with participants in the lowest quartile, participants in the highest quartile of total SPD had a significantly lower risk of CVD mortality (HR = 0.68, 95% CI: 0.51–0.91) and all-cause mortality (HR = 0.70, 95% CI: 0.60–0.82); participants in the highest tertiles or quartiles of vegetable-derived SPD, cereal-derived SPD, legume-derived SPD, nut-derived SPD, and cheese-derived SPD had a lower risk of CVD mortality (HR _vegetable − derivedSPD_ = 0.68, 95% CI: 0.54–0.86; HR _cereal − derivedSPD_ = 0.75, 95% CI: 0.57–0.97; HR _legume − derivedSPD_ = 0.68, 95% CI: 0.52–0.88; HR _nut − derivedSPD_ = 0.66, 95% CI: 0.53–0.80; HR _cheese − derivedSPD_ = 0.68, 95% CI: 0.52–0.88) and all-cause mortality (HR _vegetable − derivedSPD_ = 0.73, 95% CI: 0.64–0.84; HR _cereal − derivedSPD_ = 0.80, 95% CI: 0.69–0.93; HR _legume − derivedSPD_ = 0.70, 95% CI: 0.60–0.80;HR _nut − derivedSPD_ = 0.72, 95% CI: 0.64–0.81; HR _cheese − derivedSPD_ = 0.70, 95% CI: 0.61–0.81) than those in the lowest tertiles or quartiles. Moreover, subgroup analysis showed consistent associations among the people with hypertension and hyperlipidemia.

**Conclusion:**

Higher intake of dietary SPD is associated with decreased risk of CVD and all-cause mortality, and among specific food origin SPD, SPD derived from vegetables, cereals, legumes, nuts, and cheese was associated with reduced CVD and all-cause mortality.

## Introduction

The rising prevalence of cardiovascular disease (CVD) has become the leading cause of morbidity and mortality worldwide that reduces human life expectancy and causes a heavy burden on the healthcare system (1). Increasing evidence establishes that nutritional factors are strongly associated with the development, treatment, and prevention of CVD (2, 3). Spermidine (SPD), as a naturally occurring endogenous polyamine, is widely available in foods of both animal and plant origins and plays a crucial role in the growth and development of eukaryotic cells (4). Increasing epidemiologic research has demonstrated that SPD has a wide range of beneficial effects, such as cardiovascular protection, immune system regulation, and neuroprotective effects (5–7).

Numerous mechanism studies have indicated that SPD could exhibit remarkable cardiac and vascular protection in a variety of model organisms by stimulating autophagy as well as anti-inflammatory and anti-oxidative stress pathways. For example, feeding SPD reduced myocardial hypertrophy and improved cardiomyocyte elasticity in aged mice *via* enhancing arterial expression of autophagy markers (8); significantly decreased infarct size and alleviated myocardial hypertrophy in SD rats by increasing autophagic flux (9); alleviated atherosclerosis by reducing epithelial fat accumulation in APOE model mice (10); and downregulated endoplasmic reticulum stress signaling components in mice with kidney injury (11).

With increasing mechanism evidence of the beneficial effects of SPD, greater attention has been paid to its life span-extending effects, which have been well demonstrated across species including yeast, nematodes, flies, mice, and rats (12–14). However, we cannot conclude that its beneficial effects are applicable to the general population because research on the relationship between SPD and survival time has been mostly limited to animal models with no epidemiological validation. Therefore, we proposed a hypothesis that higher dietary SPD is associated with increased survival time in human beings. In this study, we examined the association between dietary SPD intake and all-cause and disease-specific mortality in the U.S. population recruited in the National Health and Nutrition Examination Survey (NHANES) from 2003 to 2014.

## Method

### Study population

NHANES is a stratified, multistage study using a nationally representative sample of the non-institutionalized civilian population of the U.S. Detailed NHANES has been provided elsewhere (15). After excluding participants with missing information on dietary SPD, all-cause and CVD mortality, and other covariates, 23,894 adults (age ≥ 18 years) with the data of interviews and examinations who participated in NHANES from 2003 to 2014, including 10,942 men and 12,952 women, were selected for this study. The NHANES protocol was approved by the National Health Statistics Research Ethics Committee, and written informed consent was obtained before data collection.

### Dietary assessment

A 24-h dietary recall survey was used to obtain food intake on 2 non-consecutive days. The first 24-h dietary recall was conducted in-person, and the second 24-h dietary recall was conducted by telephone 3 to 10 days later. Dietary energy and nutrient intakes were estimated using the USDA Dietary Study Food and Nutrient Database. Dietary intake components were integrated into 37 MyPyramid major groups and subgroups according to the USDA MyPyramid Equivalency Database 2.0 (MPED 2.0) User's Guide for Survey Foods. The mean values of nutrient intake on the first and the second day of the 24-h dietary recall were calculated in the analysis. Dietary supplement use was obtained through a dietary supplement questionnaire.

We calculated the amount of total dietary SPD and the amount of SPD in foods of animal origin including fresh meat, cooked meat, dairy products, eggs, cheese, and seafood, and in foods of plant origin including vegetables, fruits, cereals, legumes, and nuts. The average SPD content (nmol/g) in various foods was determined based on previous research (16). The daily intake of SPD (nmol/d) was based on the average SPD content (nmol/g) and the daily intake of various food components (mg/d).

### Main exposure and main outcomes

The main exposures in our study were the accounts of total dietary SPD intake and SPD intake from foods of plant origin including vegetables, fruits, cereals, legumes, and nuts, and foods of animal origin including fresh meat, cooked meat, dairy products, eggs, cheese, and seafood. The outcome was the status of mortality as determined by the National Death Index (NDI). The NDI is a considerably reliable and widely used death identification resource. The ICD-10 was used to determine disease-specific death. ICD-10 codes I00–I09, I11, I13, I20–I51, or I60–I69 were assigned to death due to CVD. In summary, a total of 736 deaths due to CVD and 2,365 deaths due to all-cause were documented.

### Covariates

The covariates in our study included age (years), sex (male/female), race/ethnicity (non-Hispanic white/Mexican American/non-Hispanic black/other), Alternative Healthy Eating Index (AHEI), current smoker (yes or no, a current smoker was defined as someone who had smoked 100 cigarettes in his or her lifetime and reported currently smoking), current drinker (having at least 12 alcohol drinks per year or not), education level (less than high school education, high school, or above), annual household income (<$20 000, ≥$20 000 and <$45 000, ≥$45 000 and <$75 000, ≥$75 000 and <$100 000, or ≥$100 000), total energy intake from the 24-h dietary recall (kcal/d), body mass index (BMI, kg/m^2^), regular exercise (having engaged in recreational moderate and vigorous physical activity (MVPA) in the past 30 days or no), and a history of hypertension, diabetes, or hyperlipidemia defined as a physician diagnosis of self-reported hypertension, diabetes, or hyperlipidemia.

### Statistical analysis

Demographic, dietary nutrient intake, and anthropometric characteristics were presented using the mean and standard deviation for the continuous variables, and number and percentage for categorical variables. The baseline characteristics were analyzed using chi-square tests and generalized linear models adjusted for age, and gender. All statistical analyses were performed by R 4.1.2 software, and the two-sided P < 0.05 was regarded as statistically significant.

### Cox proportional hazard (CPH) models

CPH models were used to calculate hazard ratios (HRs) and 95% CI for all-cause and CVD mortality. The time scale in the Cox model used the follow-up time obtained by person-months from the date of the interview to their death, or the end of 2015. The dietary SPD was categorized into quartiles and tertiles, and the lowest quartile and tertile are regarded as the reference group. The confounders in the CPH model included age, sex, race, smoking status, drinking status, exercise, total energy intake, education level, energy intake, annual household income, BMI, AHEI, diabetes, hypertension, and hyperlipidemia. We performed a log transformation of all non-normal continuous variables. The gender interaction in the Cox proportional hazard (CPH) model was conducted.

### Sensitivity analysis

A total of five sets of sensitivity analyses were performed in this study. In sets 1 to set 2, we analyzed the relationship between dietary SPD and all-cause and CVD mortality in the hypertension population and hyperlipidemia population, respectively, to identify the robustness of our results. In set 3 and set 4, we analyzed the relationship between dietary SPD and all-cause and CVD mortality in male and female populations separately. The participants who had a follow-up time <5 years were analyzed in set 5.

## Result

### Baseline characteristics

The demographic and nutritional characteristics of the participants are presented in Table 1. Compared with survivors, the participants with CVD and all-cause mortality were more likely to be male, older, and non-Hispanic whites; have a higher prevalence of hypertension, diabetes, and hyperlipidemia; have lower BMI, household income, education level, and total energy intake; and more prone to have a higher intake of dairy-derived SPD and a lower intake of total SPD, vegetable-derived SPD, legume-derived SPD, fresh meat-derived SPD, nut-derived SPD, and cheese-derived SPD (all *P* < 0.05).

**Table 1:** Baseline characteristics of variables in survived people, CVD mortality, and all-cause mortality status.

**Variable**	**Survival–people (*N =* 21,529)**	**CVD mortality** **(*N =* 736)**	***P*** **–value (Survival people vs. CVD mortality)**	**All–cause mortality** **(*N =* 2,365)**	***P*** **–value (Survival people vs. all–cause mortality)**
Age (years)	49.0[36.0–63.0]	76.0[66.0–80.0]	<0.001	75.0 [64.0–80.0]	<0.001
Male, *N* (%)	10,026.0 (46.6%)	434.0 (59.0%)	<0.001	1,350.0 (57.1%)	<0.001
Non–Hispanic white, *N* (%)	10,188.0 (47.3%)	467.0 (63.5%)	<0.001	1,484.0 (62.7%)	<0.001
College graduate or above, *N* (%)	5,437.0 (25.3%)	99.0 (13.5%)	<0.001	315.0 (13.3%)	<0.001
>$100,000 annual household income, *N* (%)	2,924.0 (13.6%)	16.0 (2.2%)	<0.001	64.0 (2.7%)	<0.001
BMI (kg/m^2^)	28.2 [24.5–32.7]	27.9[24.5–31.7]	0.083	27.4 [24.1–31.7]	<0.001
Total energy intake (kcal/d)	1,912.5 [1,469.0–2,473.0]	1,577.8[1,235.6–1,990.2]	<0.001	1,633.0 [1,274.0–2,058.0]	<0.001
Regular exercise, *N* (%)	5,143.0 (23.9%)	153.0 (20.8%)	0.2	479.0 (20.3%)	<0.001
Current drinking, *N* (%)	14,506.0 (67.4%)	447.0 (60.7%)	0.004	1,437.0 (60.8%)	<0.001
Current smoking, *N* (%)	4,659.0 (21.6%)	126.0 (17.1%)	0.023	468.0 (19.8%)	0.06
Hypertension, *N* (%)	8,055.0 (37.4%)	510.0 (69.3%)	<0.001	1,511.0 (63.9%)	<0.001
Hyperlipidemia, *N* (%)	8,239.0 (38.3%)	393.0 (53.4%)	<0.001	1,198.0 (50.7%)	<0.001
Diabetes, *N* (%)	2,622.0 (12.2%)	216.0 (29.3%)	<0.001	626.0 (26.5%)	<0.001
Total SPD (μm/d)	378.6 [271.2–512,5]	304.6 [228.0.5–401.1]	<0.001	316.5 [235.2–419.7]	<0.001
Fruit SPD (μm/d)	4.2[0.7–8.7]	4.9[1.4–8.7]	0.056	4.6[1.4–8.8]	0.007
Vegetable SPD (μm/d)	20.1[12.0–31.1]	16.9 [9.5–26.0]	<0.001	17.8[9.9–27.6]	<0.001
Cereals SPD (μm/d)	309.2[212.2–427.5]	254.6[189.6–348.4]	<0.001	264.9[189.9–358.4]	<0.001
Legumes SPD (μm/d)	8.2[0,6–18.7]	2.9[0.0–10.2]	<0.001	3.3[0.0–11.0]	<0.001
Fresh meat SPD (μm/d)	3.2[1.6–5.5]	2.6[1.2–4.2]	<0.001	2.6[1.1–4.3]	<0.001
Cooked meat SPD (μm/d)	5.6[3.4–8.5]	4.5[2.7–6.8]	<0.001	4.5[2.7–7.1]	<0.001
Nuts SPD (μm/d)	0.0 [0.0–3.5]	0.0[0.0–1.9]	<0.001	0.0 [0.0–2.0]	<0.001
Egg SPD (μm/d)	0.07[0.01–0.3]	0.08[0.01–0.3]	0.9	0.07[0.01–0.3]	0.4
Seafood SPD (μm/d)	0.0[0.0–1.7]	0.0[0.0–0.5]	0.002	0.0[0.0–0.8]	<0.001
Milk&Yogurt SPD (μm/d)	0.2[0.1–0.5]	0.3[0.1–0.6]	<0.001	0.3[0.1–0.6]	<0.001
Cheese SPD (μm/d)	9.0[0.7–20.8]	3.3[0.0–11.4]	<0.001	3.6[0.0–12.2]	<0.001

Continuous variables are presented as medians (IQRs). Categorical variables are presented as a percentage.

### Dietary SPD and mortality

**Figure 1:**
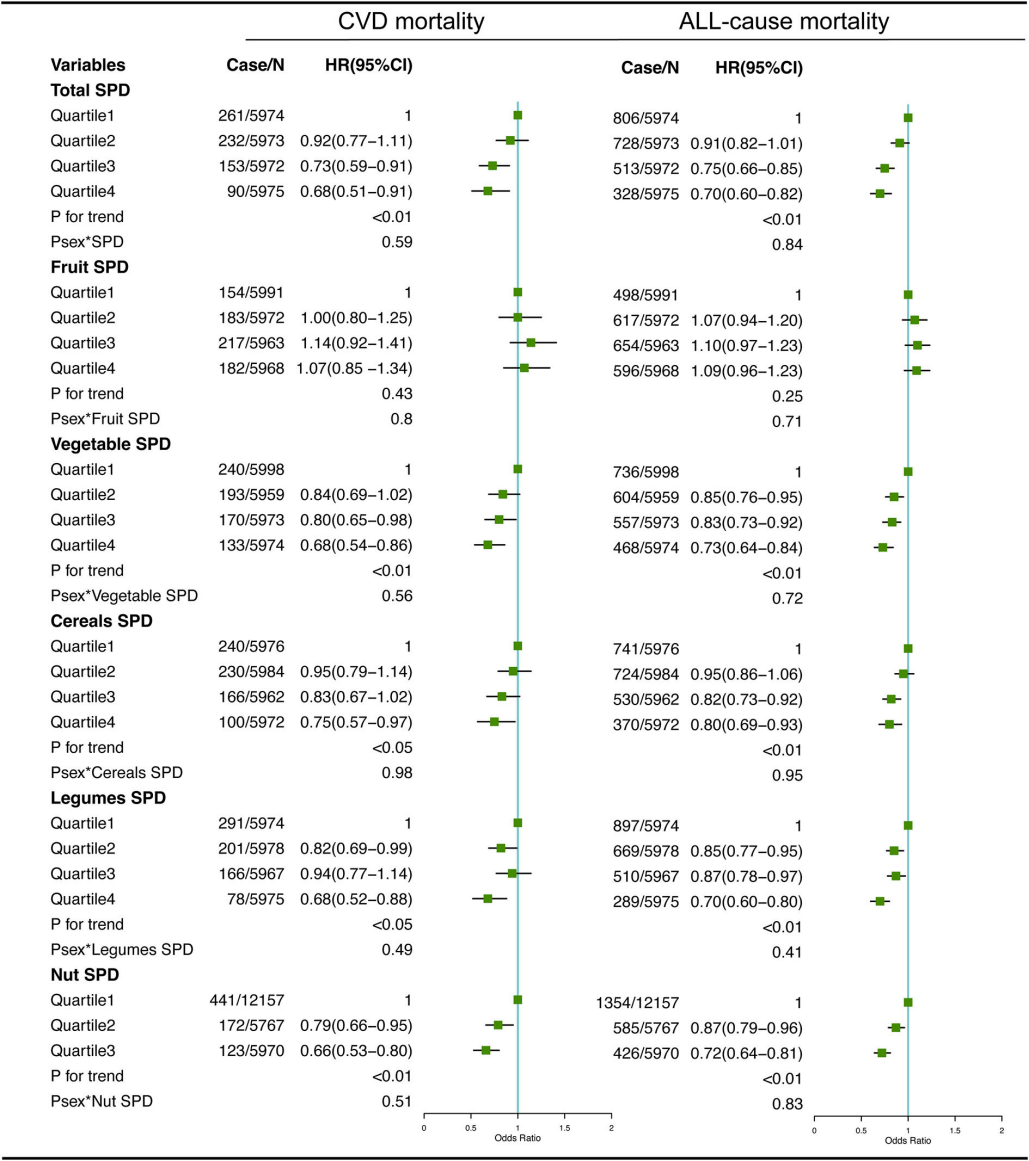
Multivariate adjusted hazard ratios (HRs) of the dietary total SPD, fruit-derived SPD, vegetable-derived SPD, cereal-derived SPD, legume-derived SPD, and nut-derived SPD with CVD and all-cause mortality. A logarithmic transformation was performed for non-normal continuous variables. Adjusting factors included age, gender, race, income, education level, regular exercise, smoking, alcohol consumption, BMI, body mass index; total energy intake, AHEI, Alternative Healthy Eating Index; diabetes, hypertension, and hyperlipidemia.

**Figure 2:**
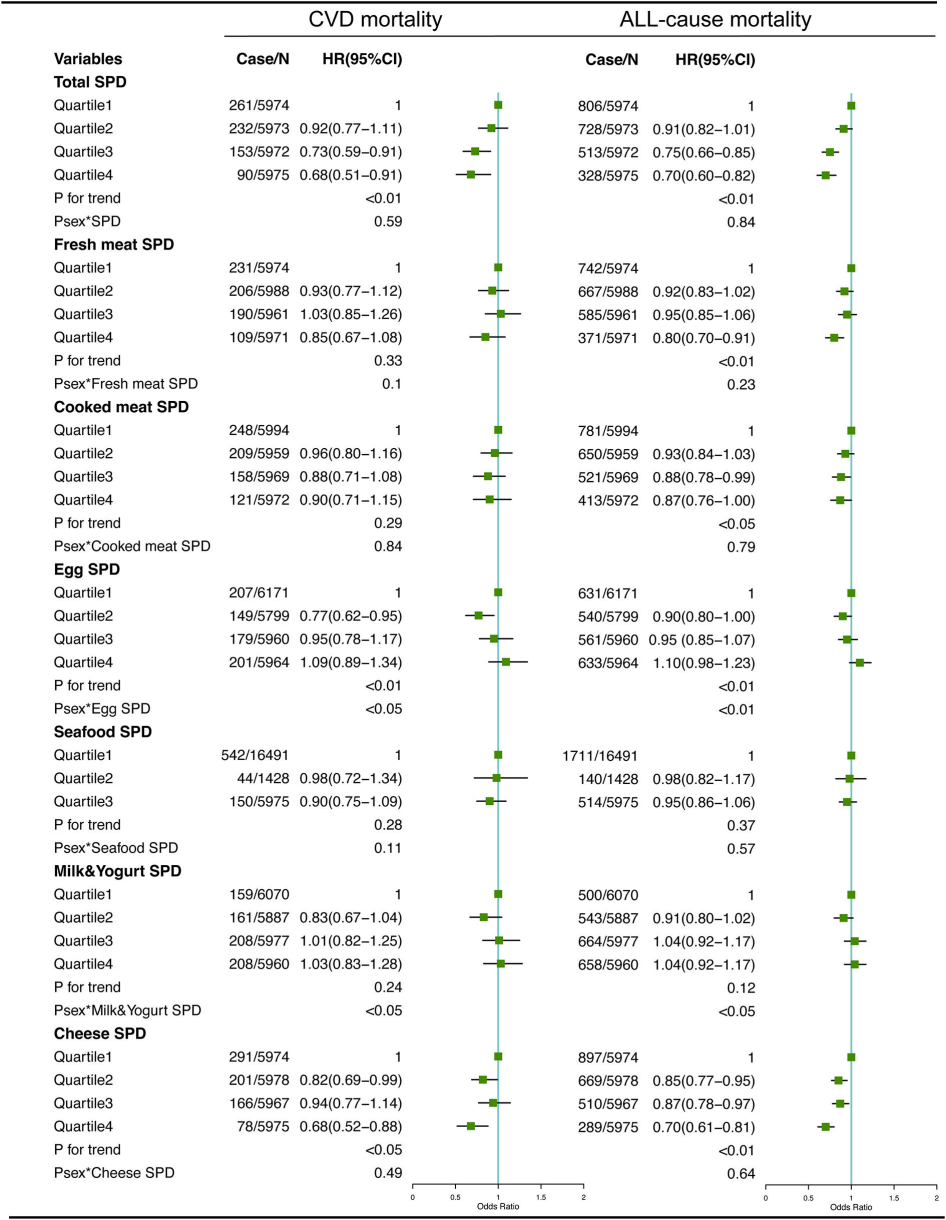
Multivariate adjusted hazard ratios (HRs) of the dietary total SPD, fresh meat-derived SPD, cooked meat-derived SPD, egg-derived SPD, seafood-derived SPD, milk & yogurt-derived SPD, and cheese-derived SPD with CVD and all-cause mortality. A logarithmic transformation was performed for non-normal continuous variables. Adjusting factors included age, gender, race, income, education level, regular exercise, smoking, alcohol consumption, BMI, body mass index; total energy intake, AHEI, Alternative Healthy Eating Index; diabetes, hypertension, and hyperlipidemia.

The associations between dietary total SPD and specific food-derived SPD intake with all-cause and CVD mortality in the total population are presented in Figures 1, 2. As indicated by HR and 95% CI, the participants in the highest quartiles (quartile 4) of total SPD had a lower risk of CVD mortality (HR = 0.68, 95% CI: 0.51–0.91) and all-cause mortality (HR = 0.70, 95% CI: 0.60–0.82) than those in the lowest quartile (quartile 1). Also, the intake of SPD from four specific food sources (vegetables, cereals, legumes, nuts, and cheese) was significantly associated with mortality outcomes.

For different dietary sources of SPD, the participants in the highest quartiles or tertiles had a lower risk of CVD mortality (HR = 0.68, 95% CI: 0.54–0.86 for vegetable-derived SPD; HR = 0.75, 95% CI: 0.57–0.97 for cereal-derived SPD; HR = 0.68, 95% CI: 0.52–0.88 for legume-derived SPD; HR = 0.66, 95% CI: 0.53–0.80 for nut-derived SPD; HR = 0.68, 95% CI: 0.52–0.88 for cheese-derived SPD) and all-cause mortality (HR = 0.73, 95% CI: 0.64–0.84 for vegetable-derived SPD; HR = 0.80, 95% CI: 0.69–0.93 for cereal-derived SPD; HR = 0.70, 95% CI: 0.60–0.80 for legume-derived SPD; HR = 0.72, 95% CI: 0.64–0.81 for nut-derived SPD; HR = 0.70, 95% CI: 0.61–0.81 for cheese-derived SPD). Sex was not a significant effect modifier of the aforementioned association (*P*_effect modification with sex_ > 0.05).

### Sensitivity analysis

Consistent with the results of the total sample, analysis of subgroups among people with hypertension (Supplemental Figures 1, 2) and hyperlipidemia (Supplemental Figures 3, 4), as well as men (Supplemental Figures 5, 6), women (Supplemental Figures 7, 8), and those with less than 5 years of follow-up (Supplemental Figures 9, 10), also showed negative associations between the total and specific food source SPD intake and CVD and all-cause mortality, which indicated our results were relatively robust.

## Discussion

To the best of our knowledge, this study is the first epidemiologic study to assess the association between dietary SPD intake and all-cause and CVD mortality among the U.S. adult population. The results of this study showed that the consumption of total dietary SPD, vegetable-derived SPD, cereal-derived SPD, legume-derived SPD, nut-derived SPD, and cheese-derived SPD was associated with decreased risk of all-cause and CVD mortality. In addition, these associations were relatively robust, which could be consistently observed among different subgroups.

Currently, increasing experimental evidence has demonstrated the autophagy, anti-inflammatory, and anti-oxidative stress effects of SPD treatment. In addition, previous studies have indicated that SPD could prolong the life span of different species, from yeast to rodents, and promote the manifestation of age-related diseases *via* the induction of protective autophagy (12). However, very few epidemiological studies have found that dietary SPD may increase the survival time in the adult population, which is the most important finding of this study. Several studies have established an inverse association between dietary SPD and the risk of cancer-specific mortality, which could partially support our findings. In line with our epidemiological analysis finding, a prospective, population-based cohort study showed that dietary SPD intake was negatively linked to the prevalence of CVD. Moreover, animal studies also corroborated the cardiovascular protective effect of SPD. An aging mice model has revealed that SPD feeding could reduce myocardial hypertrophy and improve aging-related cardiomyocyte elasticity (17). Another animal study showed that SPD significantly reduced the infarct size of myocardial infarction in SD rats by increasing autophagic flux through the AMPK/mTOR signaling pathway and alleviated myocardial hypertrophy (9), and SPD-induced autophilia could prevent atherosclerosis by reducing epithelial fat accumulation in vascular smooth muscle cells (VSMCs) of the APOE model mice (10), alleviate vascular calcification in kidney injury (11), reverse aging-related vascular calcification, and improve aging-reduced aortic elasticity (8). These studies argued for autophagy as a pivotal mechanism underlying SPD-induced cardioprotection.

Autophagy is a complex degradation/recycling system in charge of non-apoptotic cell death and intracellular degradation of misfolded or aggregated proteins and dysfunctional organelles such as mitochondria, which is essential for maintaining cellular renovation and homeostasis (7, 18, 19). The role of SPD-induced autophagy has been widely demonstrated to be essential not only for cardiovascular system protection but also for alleviation of age-related cognitive impairment, and life span extension (7, 20, 21). Interestingly, autophagy and serum SPD in humans and multiple model organisms showed a significant age-related decrease (8). However, the prevalence of CVD dramatically increases with age (22). This has led to the conjecture that the autophagic capacity decreased with age, leading to a large accumulation of damaged cells and dysfunctional intracellular organelles in the cardiovascular system, thus causing CVD. However, intake of more dietary SPD and SPD-induced autophagy reversed this process, resulting in cardioprotective effects. This mechanism evidence may strongly support the negative association of dietary SPD with the reduced risk of CVD mortality.

In addition to the induction of autophagy, several potential mechanisms for the cardioprotective effects of SPD may exist as follows. As known, oxidative damage and inflammation are widely reported as the main factors leading to atherosclerosis, which is the most typical and major pathological change of CVD. The accumulation of oxidized LDL in the vascular endothelium is the most important factor during early plaque formation in atherosclerosis (23, 24), and the aggregation of pro-inflammatory factors may contribute to the acceleration of vascular calcification. As the most important member of the polyamine family, SPD is synthesized from putrescine and serves as a precursor of spermine (25). Numerous *in vitro* and *in vivo* experiments have shown that SPD and spermine may act as scavengers of ROS and then protect the cardiovascular system from oxidative damage (26). SPD has been linked to increased titin phosphorylation, which inhibits downstream inflammation and thus increases cardiomyocyte elasticity (17). In addition, due to its polycationic nature, SPD can readily bind negatively charged biological macromolecules, including DNA, RNA, proteins, and phospholipids, and can modulate the function of these macromolecules in many cases. It has been shown that SPD can enhance the stability and flexibility of DNA (26), which may be an underlying mechanism for the cardioprotective effect of SPD. Moreover, arginine, the raw material for SPD synthesis *in vivo*, is a substrate for the synthesis of nitric oxide (NO), which is a recognized cardiovascular dilator (27). NO also showed an age-related decrease (28, 29). It has been shown that SPD could improve the bioavailability of arginine for NO synthesis, implying that relatively sufficient SPD can lead to greater conversion of arginine to NO, thereby protecting the cardiovascular system (11, 12). This is another potential mechanism for the cardioprotective effect of SPD. In our study, these beneficial effects were found to be relatively robust, which was significant across many subgroups, including different genders and disease states, suggesting that a higher intake of dietary SPD contributes to a cardiovascular protective effect. Furthermore, significant sex-mediated effects were not found, except for the egg-derived SPD.

Another key finding of this study was the negative association between SPD from a specific food origin and CVD mortality. Our results suggest that vegetables, cereals, legumes, nuts, and cheese would be better sources of SPD to protect the cardiovascular system. SPD is widely found in all foods containing nucleic acids and is abundant in coarse cereals, wheat germ, vegetables, and fermented foods containing bacteria and fungi such as stinky cheese and natto (16, 30). We found that dietary intake of SPD from vegetables, cereals, legumes, nuts, and cheese sources could significantly reduce the risk of CVD mortality. These dietary SPD sources with cardioprotective effects coincided with the Mediterranean dietary pattern, which is a recognized dietary pattern with proven cardiovascular protective effects (31). Although the negative relationships between the consumption of vegetables, cereals, legumes, nuts, and cheese and CVD mortality have been well established (32–35), the association between dietary intake of SPD from the aforementioned foods and CVD mortality was still significant after adjusting for AHEI, which indicates the protective role of dietary SPD was independent of other beneficial ingredients in food. Therefore, vegetables, cereals, legumes, nuts, and cheese should be consumed in greater quantities as an ideal food source of cardiovascular protective SPD. In addition, SPD is a highly absorbable polyamine that could be taken up in the small intestine and utilized by multiple systems without secondary degradation in the circulatory system, which allows dietary SPD to significantly contribute to elevated SPD concentrations in the cardiovascular system (36, 37). That is also an underlying biological basis for the cardioprotective effects of dietary SPD.

This study has several strengths. First, this is the first epidemiologic study to evaluate the relationship between dietary SPD and CVD mortality in a representative population sample of U.S. adults. Second, we identified specific dietary sources of SPD with cardio protective effects, which provide guidance for dietary supplementation of SPD. Third, the association reported in this study was relatively robust that it was significant in a multitude of subgroups so that we could provide dietary supplementation strategies for SPD in gender-specific populations, as well as in populations with multiple chronic diseases. We are also aware that this study has certain limitations. First, although 24-h dietary recall is the most valid and universal method of investigating dietary information in observational studies, measurement error arising from day-to-day variation in food intake still exists. Second, we were unable to control for variables that were not measured in the observational study. Third, this study lacked an internal exposure evaluation of dietary SPD, which needs to be refined for future research.

In conclusion, a higher intake of dietary SPD is associated with decreased risk of CVD and all-cause mortality, and among specific food origin SPD, vegetable-, cereal-, legume-, nut-, and cheese-derived SPD was associated with reduced CVD and all-cause mortality.

## Data availability statement

Publicly available datasets were analyzed in this study. This data can be found here: https://www.cdc.gov/nchs/nhanes/index.htm.

## Author contributions

CS, TH, and WJ conceived the idea. TH and WW drafted the manuscript. HW, WJ, and HJ conducted data interpretation. CH, JW, and ZW conducted the first analysis. YC, WJ, XL, and XS conducted the second analysis for verification. All authors critically assessed, reviewed and approved the manuscript.

## Funding

This research was supported by funds from HMU Marshal Initiative Funding (HMUMIF-21011 to WJ; HMUMIF-21013 to WW).

## Conflict of interest

The authors declare that the research was conducted in the absence of any commercial or financial relationships that could be construed as a potential conflict of interest.

## Publisher's note

All claims expressed in this article are solely those of the authors and do not necessarily represent those of their affiliated organizations, or those of the publisher, the editors and the reviewers. Any product that may be evaluated in this article, or claim that may be made by its manufacturer, is not guaranteed or endorsed by the publisher.

## Abbreviations

SPD: spermidine
CVD: cardiovascular disease
NHANES: National Health and Nutrition Examination Survey
HR: hazard ratio
NDI: National Death Index
BMI: body mass index
AHEI: Alternative Healthy Eating Index
CPH: Cox proportional hazards
NO: nitric oxide.
