# Supplementary material for: The association of dietary spermidine with all-cause mortality and CVD mortality: The U.S. National Health and Nutrition Examination Survey, 2003 to 2014
Source: Front Public Health. 2022 Sep 28;10:949170. doi: 10.3389/fpubh.2022.949170 (PMC9554131; doi:10.3389/fpubh.2022.949170)
Supplement: Supplementary file 1 [file Data_Sheet_1.PDF]

Supplemental Figure 1

Variables	CVD mortality Case/N	CVD mortality HR(95%CI)	ALL-cause mortality Case/N	ALL-cause mortality HR(95%CI)
**Total SPD**				
Quartile1	163/2392	1	465/2392	1
Quartile2	148/2391	0.87(0.70–1.10)	451/2391	0.94(0.82–1.08)
Quartile3	121/2390	0.82(0.64–1.06)	346/2390	0.78(0.67–0.91)
Quartile4	78/2393	0.70(0.51–0.97)	249/2393	0.70(0.58–0.85)
P for trend		<0.05		<0.01
Psex*SPD		0.53		0.71
**Fruit SPD**				
Quartile1	116/2404	1	348/2404	1
Quartile2	122/2390	0.96(0.74–1.24)	395/2390	1.04(0.90–1.21)
Quartile3	148/2382	1.14(0.88–1.46)	390/2382	1.00(0.86–1.16)
Quartile4	124/2390	1.02(0.78–1.33)	378/2390	1.02(0.87–1.19)
P for trend		0.71		0.97
Psex*Fruit SPD		0.89		0.46
**Vegetable SPD**				
Quartile1	150/2400	1	463/2400	1
Quartile2	140/2396	0.99(0.78–1.25)	392/2396	0.88(0.76–1.01)
Quartile3	134/2380	1.00(0.79–1.28)	374/2380	0.86(0.75–1.00)
Quartile4	86/2390	0.71(0.53–0.94)	282/2390	0.69(0.59–0.81)
P for trend		<0.05		<0.01
Psex*Vegetable SPD		0.54		0.69
**Cereals SPD**				
Quartile1	155/2397	1	446/2397	1
Quartile2	150/2386	0.91(0.72–1.14)	448/2386	0.95(0.83–1.09)
Quartile3	119/2396	0.81(0.63–1.04)	351/2396	0.81(0.69–0.93)
Quartile4	86/2387	0.74(0.55–1.00)	266/2387	0.73(0.61–0.87)
P for trend		<0.05		<0.01
Psex*Cereals SPD		0.45		0.97
**Legumes SPD**				
Quartile1	198/2583	1	548/2583	1
Quartile2	125/2221	0.80(0.63 –1.00)	384/2221	0.85(0.74–0.97)
Quartile3	117/2408	0.81(0.64–1.03)	346/2408	0.83(0.72–0.95)
Quartile4	70/2354	0.70(0.52–0.93)	233/2354	0.78(0.66–0.92)
P for trend		<0.05		<0.01
Psex*Legumes SPD		0.48		0.46
**Nut SPD**				
Quartile1	315/4973	1	889/4973	1
Quartile2	111/2246	0.74(0.59–0.93)	362/2246	0.85(0.75– 0.97)
Quartile3	84/2347	0.61(0.48–0.79)	260/2347	0.66(0.57–0.76)
P for trend		<0.01		<0.01
Psex*Nut SPD		0.51		0.9

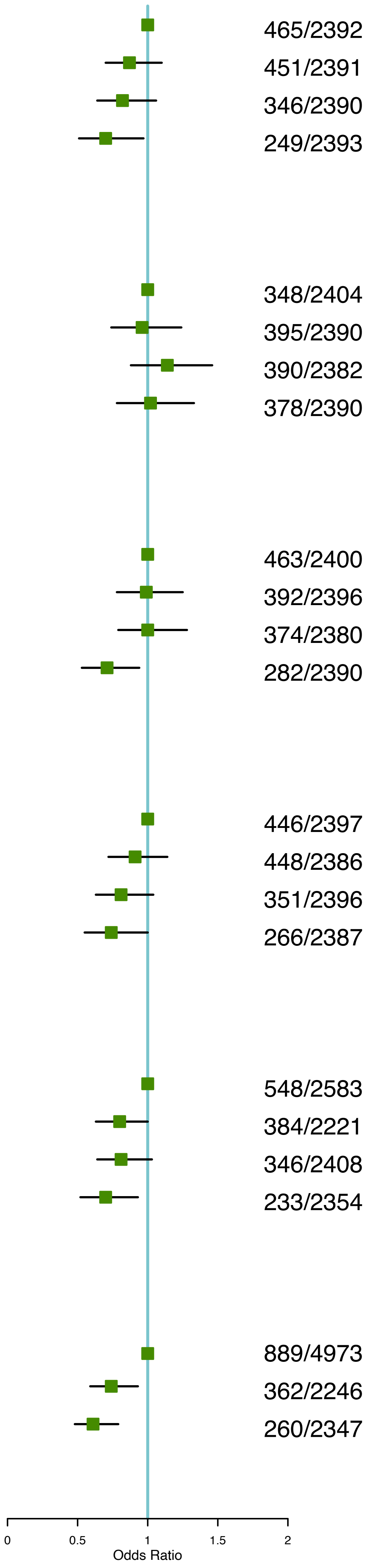

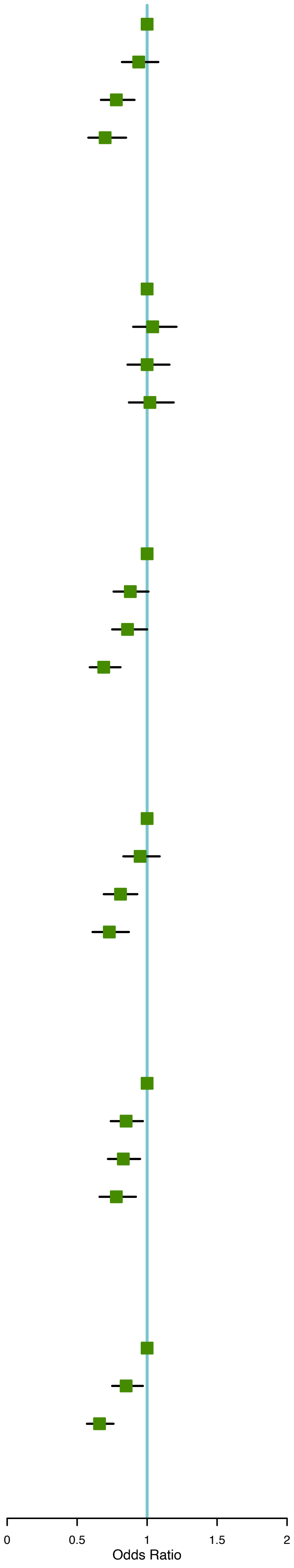

Supplemental Figure 2

Variables	CVD mortality Case/N	CVD mortality HR(95%CI)	ALL-cause mortality Case/N	ALL-cause mortality HR(95%CI)
**Total SPD**				
Quartile1	163/2392	1	465/2392	1
Quartile2	148/2391	0.87(0.70–1.10)	451/2391	0.94(0.82–1.08)
Quartile3	121/2390	0.82(0.64–1.06)	346/2390	0.78(0.67–0.91)
Quartile4	78/2393	0.70(0.51–0.97)	249/2393	0.70(0.58–0.85)
P for trend		<0.05		<0.01
Psex*SPD		0.53		0.71
**Fresh meat SPD**				
Quartile1	160/2399	1	469/2399	1
Quartile2	132/2391	0.85(0.67–1.07)	405/2391	0.88(0.77–1.00)
Quartile3	121/2385	0.91(0.71–1.16)	372/2385	0.92(0.80–1.06)
Quartile4	97/2391	0.97(0.74–1.27)	265/2391	0.81(0.70 –0.95)
P for trend		0.91		<0.05
Psex*Fresh meat SPD		0.04		0.15
**Cooked meat SPD**				
Quartile1	159/2392	1	492/2392	1
Quartile2	142/2391	1.02(0.81–1.28)	406/2391	0.93(0.81–1.06)
Quartile3	117/2391	0.98(0.76–1.26)	344/2391	0.88 (0.77–1.02)
Quartile4	92/2392	1.03(0.77–1.38)	269/2392	0.87(0.74–1.03)
P for trend		0.91		0.08
Psex*Cooked meat SPD		0.79		0.76
**Egg SPD**				
Quartile1	144/2393	1	413/2393	1
Quartile2	109/2406	0.76(0.59 –0.98)	366/2406	0.87(0.76–1.01)
Quartile3	119/2374	0.92(0.72–1.18)	334/2374	0.86(0.74–1.00)
Quartile4	138/2393	1.09(0.85–1.38)	398/2393	1.06(0.91– 1.22)
P for trend		0.08		0.09
Psex*Egg SPD		0.17		<0.05
**Seafood SPD**				
Quartile1	378/6573	1	1109/6573	1
Quartile2	34/606	1.01(0.71–1.44)	94/606	0.94(0.76–1.16)
Quartile3	98/2387	0.86(0.69–1.08)	308/2387	0.90(0.79–1.03)
P for trend		0.2		0.12
Psex*Seafood SPD		0.76		0.47
**Milk&Yogurt SPD**				
Quartile1	121/2412	1	360/2412	1
Quartile2	110/2385	0.81(0.62–1.05)	326/2385	0.80(0.69–0.93)
Quartile3	128/2376	0.90(0.70 –1.16)	383/2376	0.92(0.79–1.06)
Quartile4	151/2393	1.02(0.79–1.31)	442/2393	1.00(0.87–1.17)
P for trend		0.35		0.13
Psex*Milk&Yogurt SPD		0.08		0.06
**Cheese SPD**				
Quartile1	198/2583	1	548/2583	1
Quartile2	125/2221	0.80(0.63–1.00)	384/2221	0.85(0.74–0.97)
Quartile3	117/2408	0.81(0.64–1.03)	346/2408	0.83(0.72–0.95)
Quartile4	70/2354	0.70(0.52–0.93)	233/2354	0.78(0.66–0.92)
P for trend		<0.05		<0.01
Psex*Cheese SPD		0.48		0.46

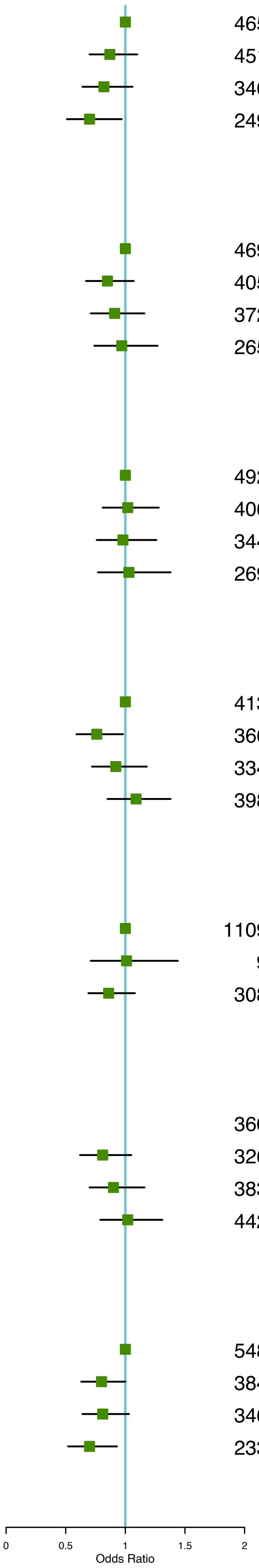

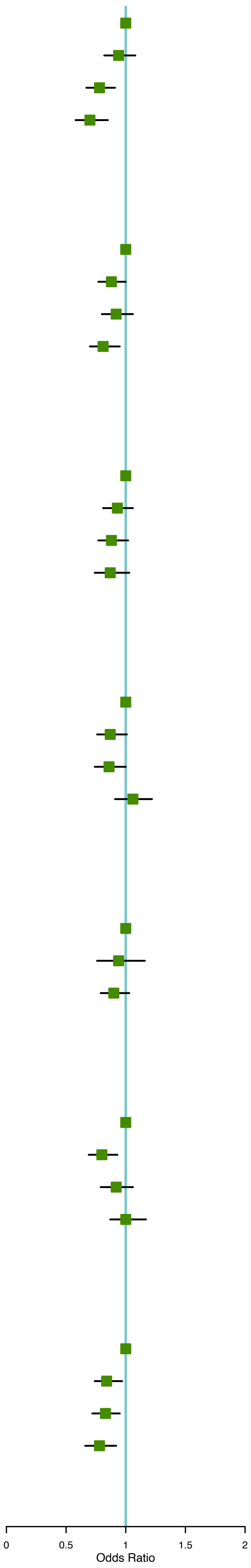

Supplemental Figure 3

Variables	CVD mortality Case/N	CVD mortality HR(95%CI)	ALL-cause mortality Case/N	ALL-cause mortality HR(95%CI)
**Total SPD**				
Quartile1	137/2360	1	384/2360	1
Quartile2	129/2358	0.99(0.77–1.27)	367/2358	0.97(0.83–1.12)
Quartile3	85/2359	0.71(0.52–0.95)	266/2359	0.75(0.63–0.89)
Quartile4	42/2360	0.49(0.33–0.73)	181/2360	0.68(0.55–0.85)
P for trend		<0.01		<0.01
Psex*SPD		0.73		0.52
**Fruit SPD**				
Quartile1	92/2360	1	282/2360	1
Quartile2	97/2360	0.98(0.73–1.31)	318/2360	1.05(0.89–1.23)
Quartile3	102/2365	1.08(0.80–1.44)	313/2365	1.05(0.89–1.24)
Quartile4	102/2352	1.20(0.90–1.62)	285/2352	1.05(0.89–1.25)
P for trend		0.14		0.63
Psex*Fruit SPD		0.16		0.3
**Vegetable SPD**				
Quartile1	128/2376	1	389/2376	1
Quartile2	116/2349	0.95(0.74–1.23)	310/2349	0.81(0.70–0.94)
Quartile3	88/2358	0.80(0.60–1.07)	271/2358	0.76(0.65–0.90)
Quartile4	61/2354	0.57(0.41–0.80)	228/2354	0.65(0.54–0.78)
P for trend		<0.01		<0.01
Psex*Vegetable SPD		1		0.09
**Cereals SPD**				
Quartile1	133/2360	1	363/2360	1
Quartile2	125/2360	0.97(0.75–1.24)	365/2360	0.99(0.86–1.15)
Quartile3	85/2362	0.72(0.54–0.96)	271/2362	0.80(0.68–0.95)
Quartile4	50/2355	0.57(0.39–0.82)	199/2355	0.75(0.61–0.92)
P for trend		<0.01		<0.01
Psex*Cereals SPD		0.53		0.84
**Legumes SPD**				
Quartile1	145/2419	1	419/2419	1
Quartile2	102/2300	0.81(0.63–1.05)	319/2300	0.86(0.74–1.00)
Quartile3	97/2362	0.99(0.76–1.29)	275/2362	0.91(0.78–1.07)
Quartile4	49/2356	0.70(0.50–0.98)	185/2356	0.79(0.66–0.95)
P for trend		0.12		<0.05
Psex*Legumes SPD		1		0.3
**Nut SPD**				
Quartile1	237/4738	1	701/4738	1
Quartile2	90/2339	0.82(0.64–1.06)	287/2339	0.85(0.74–0.98)
Quartile3	66/2360	0.72(0.55–0.96)	210/2360	0.73(0.62–0.86)
P for trend		<0.05		<0.01
Psex*Nut SPD		0.99		0.57

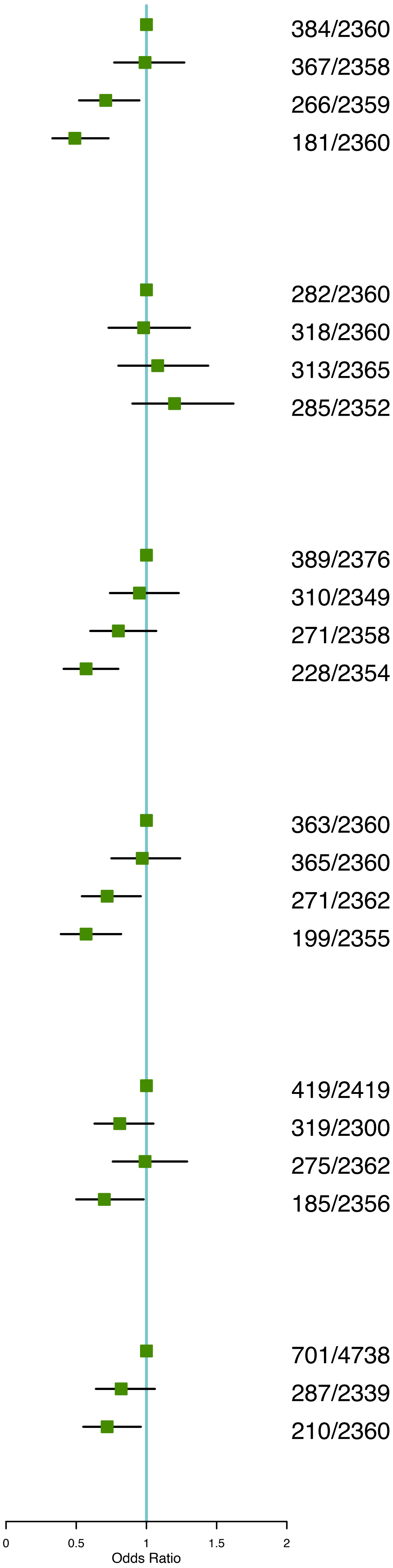

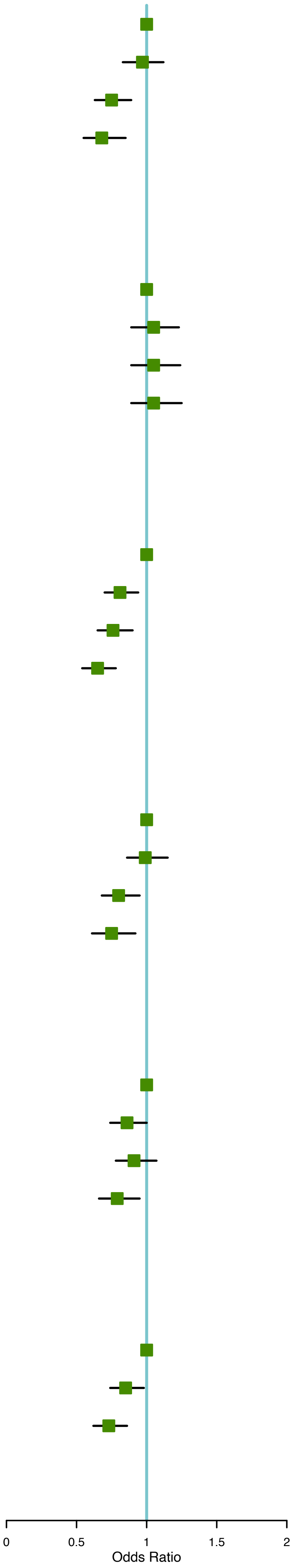

Supplemental Figure 4

Variables	CVD mortality Case/N	CVD mortality HR(95%CI)	ALL-cause mortality Case/N	ALL-cause mortality HR(95%CI)
**Total SPD**				
Quartile1	137/2360	1	384/2360	1
Quartile2	129/2358	0.99(0.77–1.27)	367/2358	0.97(0.83–1.12)
Quartile3	85/2359	0.71(0.52–0.95)	266/2359	0.75(0.63–0.89)
Quartile4	42/2360	0.49(0.33–0.73)	181/2360	0.68(0.55–0.85)
P for trend		<0.01		<0.01
Psex*SPD		0.73		0.52
**Fresh meat SPD**				
Quartile1	126/2360	1	378/2360	1
Quartile2	108/2358	0.93(0.72–1.21)	320/2358	0.91(0.78–1.05)
Quartile3	91/2360	0.90(0.68–1.18)	297/2360	0.92(0.79–1.08)
Quartile4	68/2359	0.93(0.68–1.28)	203/2359	0.80(0.66–0.96)
P for trend		0.6		<0.05
Psex*Fresh meat SPD		0.1		0.41
**Cooked meat SPD**				
Quartile1	126/2360	1	377/2360	1
Quartile2	117/2360	1.03(0.80–1.33)	328/2360	0.94(0.81–1.10)
Quartile3	87/2358	0.92(0.69–1.22)	264/2358	0.88(0.75–1.04)
Quartile4	63/2359	0.80(0.57–1.12)	229/2359	0.86(0.71–1.04)
P for trend		0.16		0.09
Psex*Cooked meat SPD		0.35		0.13
**Egg SPD**				
Quartile1	112/2459	1	321/2459	1
Quartile2	87/2267	0.86(0.65–1.15)	282/2267	0.94(0.80–1.11)
Quartile3	87/2364	0.91(0.69–1.22)	282/2364	0.99(0.84–1.16)
Quartile4	107/2347	1.18(0.90–1.56)	313/2347	1.14(0.97–1.33)
P for trend		0.07		<0.05
Psex*Egg SPD		0.03		<0.01
**Seafood SPD**				
Quartile1	290/6397	1	850/6397	1
Quartile2	26/680	0.93(0.62–1.39)	81/680	0.97(0.77–1.23)
Quartile3	77/2360	0.91(0.70–1.18)	267/2360	1.03(0.90–1.19)
P for trend		0.48		0.66
Psex*Seafood SPD		0.55		0.72
**Milk&Yogurt SPD**				
Quartile1	88/2360	1	271/2360	1
Quartile2	89/2373	0.87(0.65–1.17)	287/2373	0.92(0.78–1.09)
Quartile3	99/2353	1.00(0.75–1.34)	316/2353	1.03(0.87–1.21)
Quartile4	117/2351	1.14(0.86–1.52)	324/2351	1.00(0.85–1.19)
P for trend		0.14		0.54
Psex*Milk&Yogurt SPD		<0.05		<0.01
**Cheese SPD**				
Quartile1	145/2419	1	419/2419	1
Quartile2	102/2300	0.81(0.63–1.05)	319/2300	0.86(0.74–1.00)
Quartile3	97/2362	0.99(0.76–1.29)	275/2362	0.91(0.78–1.07)
Quartile4	49/2356	0.70(0.50–0.98)	185/2356	0.79(0.66–0.95)
P for trend		0.12		<0.05
Psex*Cheese SPD		1		0.29

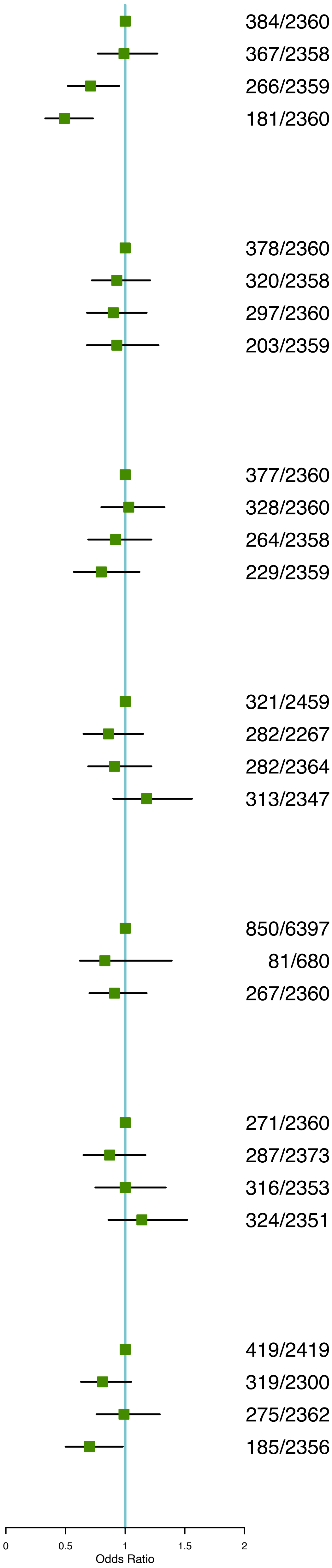

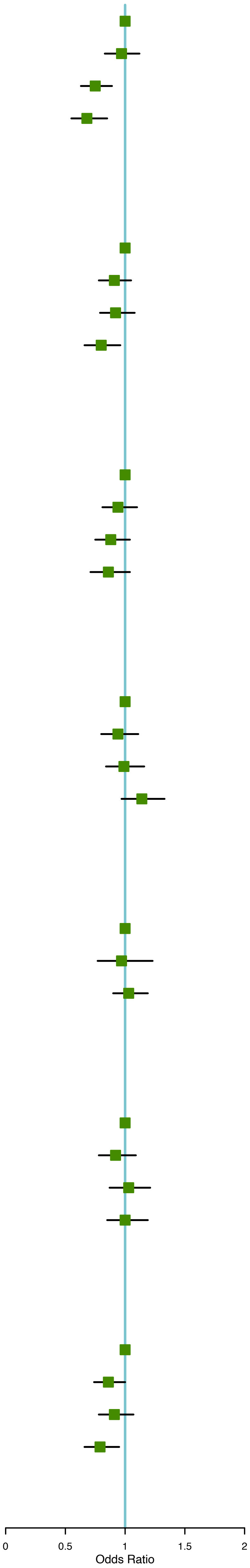

Supplemental Figure 5

Variables	CVD mortality Case/N	CVD mortality HR(95%CI)	ALL-cause mortality Case/N	ALL-cause mortality HR(95%CI)
**Total SPD**				
Quartile1	185/2844	1	545/2844	1
Quartile2	133/2844	0.76(0.60–0.96)	386/2844	0.78(0.68–0.89)
Quartile3	83/2844	0.67(0.50–0.90)	269/2844	0.69(0.58–0.81)
Quartile4	33/2844	0.40(0.26–0.61)	150/2844	0.56(0.45–0.69)
P for trend		<0.01		<0.01
**Fruit SPD**				
Quartile1	83/2848	1	273/2848	1
Quartile2	119/2840	1.25(0.94–1.67)	369/2840	1.16(0.99–1.37)
Quartile3	127/2851	1.26(0.95–1.69)	392/2851	1.17(0.99–1.37)
Quartile4	105/2837	1.25(0.92–1.69)	316/2837	1.08(0.91–1.28)
P for trend		0.35		0.86
**Vegetable SPD**				
Quartile1	150/2865	1	447/2865	1
Quartile2	108/2826	0.76(0.59–0.98)	336/2826	0.78(0.67–0.90)
Quartile3	99/2842	0.81(0.62–1.07)	313/2842	0.81(0.70–0.95)
Quartile4	77/2843	0.64(0.47–0.87)	254/2843	0.66(0.56–0.79)
P for trend		<0.01		<0.01
**Cereals SPD**				
Quartile1	166/2852	1	492/2852	1
Quartile2	135/2845	0.91(0.72–1.15)	400/2845	0.88(0.76–1.01)
Quartile3	84/2835	0.71(0.54–0.94)	276/2835	0.74(0.63–0.87)
Quartile4	49/2844	0.66(0.46–0.94)	182/2844	0.74(0.61–0.89)
P for trend		<0.01		<0.01
**Legumes SPD**				
Quartile1	177/2859	1	528/2859	1
Quartile2	116/2846	0.74(0.58–0.93)	387/2846	0.82(0.72–0.94)
Quartile3	97/2846	0.88(0.68–1.14)	278/2846	0.81(0.70–0.94)
Quartile4	44/2825	0.63(0.44–0.90)	157/2825	0.68(0.56–0.82)
P for trend		<0.05		<0.01
**Nut SPD**				
Quartile1	260/6034	1	796/6034	1
Quartile2	99/2503	0.88(0.70–1.12)	315/2503	0.89(0.78–1.02)
Quartile3	75/2839	0.70(0.54–0.92)	239/2839	0.70(0.61–0.82)
P for trend		<0.05		<0.01

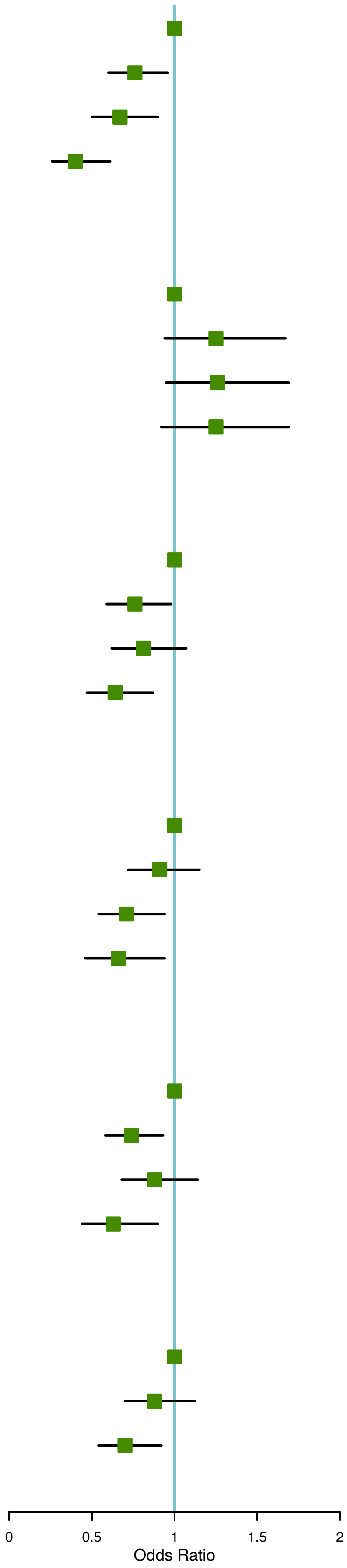

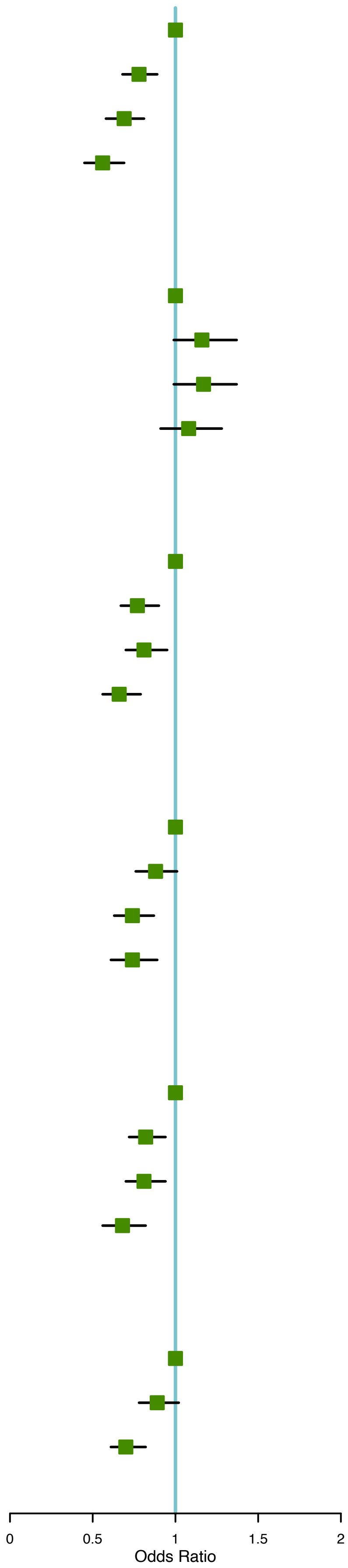

Supplemental Figure 6

Variables	CVD mortality Case/N	CVD mortality HR(95%CI)	ALL-cause mortality Case/N	ALL-cause mortality HR(95%CI)
**Total SPD**				
Quartile1	185/2844	1	545/2844	1
Quartile2	133/2844	0.76(0.60–0.96)	386/2844	0.78(0.68–0.89)
Quartile3	83/2844	0.67(0.50–0.90)	269/2844	0.69(0.58–0.81)
Quartile4	33/2844	0.40(0.26–0.61)	150/2844	0.56(0.45–0.69)
P for trend		<0.01		<0.01
**Fresh meat SPD**				
Quartile1	127/2847	1	439/2847	1
Quartile2	147/2843	1.29(1.01–1.64)	419/2843	1.04(0.91–1.19)
Quartile3	102/2847	1.14(0.87–1.49)	295/2847	0.90(0.77–1.05)
Quartile4	58/2839	1.03(0.74–1.43)	197/2839	0.90(0.76–1.08)
P for trend		0.91		0.13
**Cooked meat SPD**				
Quartile1	165/2844	1	504/2844	1
Quartile2	133/2848	1.03(0.82–1.30)	372/2848	0.92(0.80–1.06)
Quartile3	77/2841	0.80(0.60–1.06)	285/2841	0.90(0.77–1.05)
Quartile4	59/2843	0.83(0.60–1.15)	189/2843	0.77(0.64–0.93)
P for trend		0.14		<0.01
**Egg SPD**				
Quartile1	99/2883	1	308/2883	1
Quartile2	95/2805	0.97(0.73–1.30)	323/2805	1.03(0.88–1.21)
Quartile3	130/2848	1.37(1.05–1.79)	356/2848	1.15(0.99–1.35)
Quartile4	110/2840	1.30(0.98–1.72)	363/2840	1.29(1.10–1.51)
P for trend		<0.05		<0.01
**Seafood SPD**				
Quartile1	314/7953	1	974/7953	1
Quartile2	26/591	1.12(0.75–1.67)	88/591	1.21(0.97–1.51)
Quartile3	94/2832	1.02(0.80–1.29)	288/2832	0.95(0.83–1.09)
P for trend		0.92		0.45
**Milk&Yogurt SPD**				
Quartile1	85/2852	1	262/2852	1
Quartile2	92/2857	0.81(0.60–1.09)	302/2857	0.87(0.74–1.03)
Quartile3	127/2833	1.09(0.82–1.45)	405/2833	1.12(0.95–1.31)
Quartile4	130/2834	1.19(0.89–1.59)	381/2834	1.12(0.94–1.32)
P for trend		<0.05		<0.05
**Cheese SPD**				
Quartile1	177/2859	1	528/2859	1
Quartile2	116/2846	0.74(0.58–0.93)	387/2846	0.82(0.72–0.94)
Quartile3	97/2846	0.88(0.68–1.14)	278/2846	0.81(0.70–0.94)
Quartile4	44/2825	0.63(0.44–0.90)	157/2825	0.69(0.56–0.82)
P for trend		<0.05		<0.01

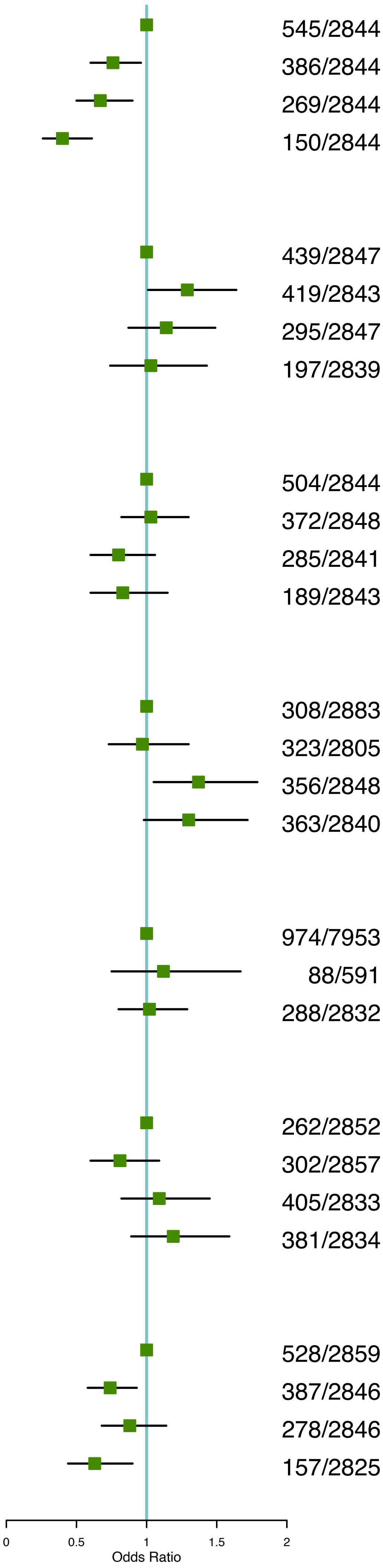

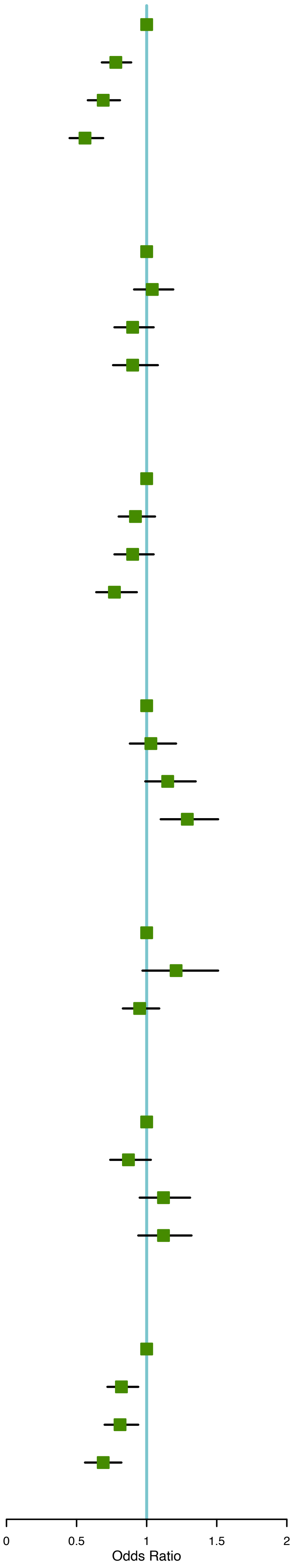

Supplemental Figure 7

Variables	CVD mortality Case/N	CVD mortality HR(95%CI)	ALL-cause mortality Case/N	ALL-cause mortality HR(95%CI)
**Total SPD**				
Quartile1	114/3130	1	345/3130	1
Quartile2	93/3129	0.85(0.64–1.12)	303/3129	0.90(0.77–1.05)
Quartile3	61/3128	0.71(0.51–0.99)	237/3128	0.88(0.74–1.05)
Quartile4	34/3131	0.74(0.47–1.15)	130/3131	0.77(0.61–0.98)
P for trend		<0.05		<0.05
**Fruit SPD**				
Quartile1	54/3131	1	200/3131	1
Quartile2	76/3160	0.99(0.69–1.41)	267/3160	1.04(0.87–1.26)
Quartile3	93/3103	1.19(0.84–1.68)	269/3103	1.04(0.86–1.26)
Quartile4	79/3124	1.09(0.76–1.56)	219/3124	1.15(0.95–1.39)
P for trend		0.52		0.13
**Vegetable SPD**				
Quartile1	93/3131	1	293/3131	1
Quartile2	82/3129	0.91(0.67–1.23)	269/3129	0.94(0.80–1.12)
Quartile3	80/3129	0.93(0.68–1.27)	262/3129	0.96(0.81–1.15)
Quartile4	47/3129	0.64(0.44–0.94)	191/3129	0.80(0.65–0.97)
P for trend		<0.05		<0.05
**Cereals SPD**				
Quartile1	79/3135		322/3135	
Quartile2	109/3130	1.14(0.86–1.50)	305/3130	0.95(0.81–1.12)
Quartile3	54/3124	0.65(0.46–0.92)	234/3124	0.84(0.70–1.00)
Quartile4	42/3129	0.90(0.60–1.35)	154/3129	0.86(0.69–1.06)
P for trend		0.14		0.07
**Legumes SPD**				
Quartile1	115/3180	1	373/3180	1
Quartile2	89/3085	0.90(0.68–1.19)	289/3085	0.85(0.73–0.99)
Quartile3	69/3135	1.03(0.76–1.40)	227/3135	0.94(0.79–1.11)
Quartile4	29/3118	0.75(0.49–1.15)	126/3118	0.79(0.64–0.98)
P for trend		0.34		0.09
**Nut SPD**				
Quartile1	189/6308	1	580/6308	1
Quartile2	64/3088	0.63(0.47–0.84)	250/3088	0.82(0.70–0.95)
Quartile3	49/3122	0.61(0.44–0.85)	185/3122	0.75(0.63–0.90)
P for trend		<0.01		<0.01

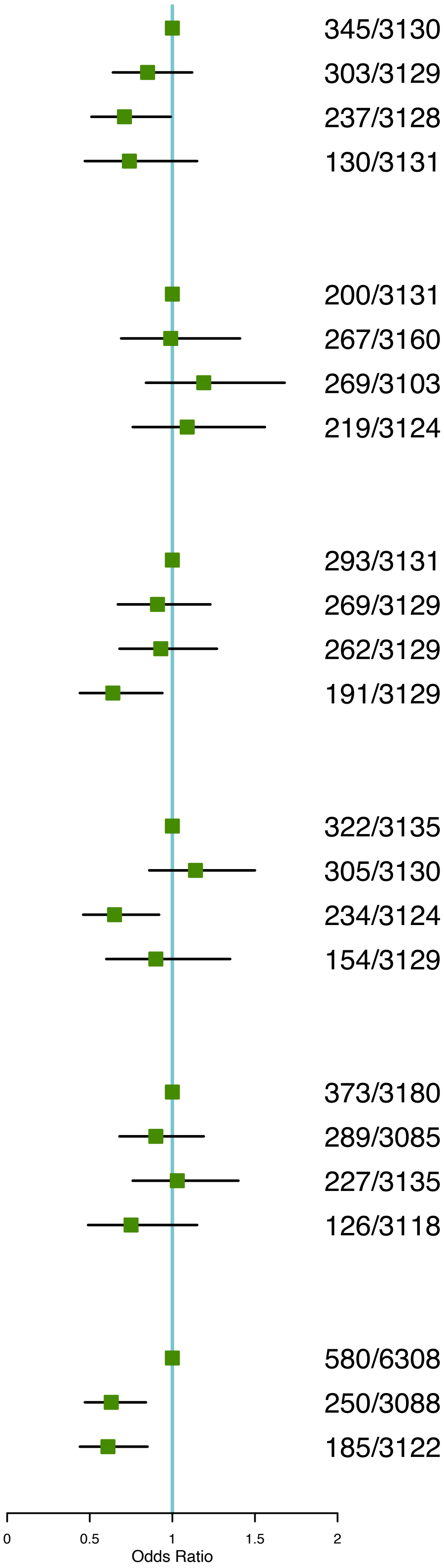

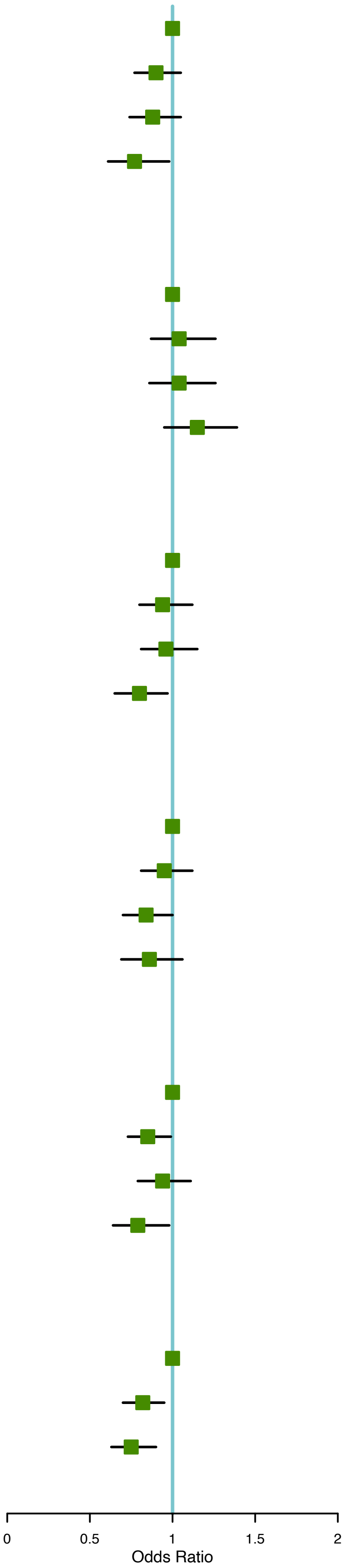

Supplemental Figure 8

	CVD mortality		ALL-cause mortality	
**Variables**	**Case/N**	**HR(95%CI)**	**Case/N**	**HR(95%CI)**
**Total SPD**				
Quartile1	114/3130	1	345/3130	1
Quartile2	93/3129	0.85(0.64–1.12)	303/3129	0.90(0.77–1.05)
Quartile3	61/3128	0.71(0.51–0.99)	237/3128	0.88(0.74–1.05)
Quartile4	34/3131	0.74(0.47–1.15)	130/3131	0.77(0.61–0.98)
P for trend		<0.05		<0.05
**Fresh meat SPD**				
Quartile1	101/3130	1	337/3130	1
Quartile2	90/3137	0.90(0.67–1.19)	258/3137	0.76(0.65–0.90)
Quartile3	68/3120	0.87(0.64–1.20)	256/3120	0.95(0.80–1.12)
Quartile4	43/3131	0.80(0.55–1.15)	164/3131	0.80(0.66–0.97)
P for trend		0.21		0.09
**Cooked meat SPD**				
Quartile1	108/3140		344/3140	
Quartile2	82/3123	0.91(0.68–1.21)	293/3123	0.98(0.83–1.14)
Quartile3	64/3124	0.88(0.64–1.21)	213/3124	0.85(0.71–1.01)
Quartile4	48/3131	0.95(0.66–1.37)	165/3131	0.87(0.72–1.07)
P for trend		0.7		0.09
**Egg SPD**				
Quartile1	105/3214	1	313/3214	1
Quartile2	53/3045	0.58(0.42–0.82)	226/3045	0.81(0.68–0.96)
Quartile3	85/3152	0.84(0.62–1.12)	264/3152	0.86(0.73–1.02)
Quartile4	59/3107	0.75(0.54–1.05)	212/3107	0.85(0.71–1.02)
P for trend		0.55		0.34
**Seafood SPD**				
Quartile1	228/8538	1	737/8538	1
Quartile2	21/851	0.96(0.61–1.50)	58/851	0.81(0.62–1.07)
Quartile3	53/3129	0.72(0.53–0.98)	220/3129	0.93(0.80–1.09)
P for trend		<0.05		0.41
**Milk&Yogurt SPD**				
Quartile1	72/3130	1	223/3130	1
Quartile2	73/3165	0.87(0.63–1.21)	253/3165	0.95(0.80–1.14)
Quartile3	77/3099	0.86(0.62–1.20)	256/3099	0.92(0.77–1.11)
Quartile4	80/3124	0.89(0.64–1.23)	273/3124	0.97(0.81–1.16)
P for trend		0.63		0.83
**Cheese SPD**				
Quartile1	115/3180	1	373/3180	1
Quartile2	89/3085	0.90(0.68–1.19)	289/3085	0.85(0.73–0.99)
Quartile3	69/3135	1.03(0.76–1.40)	227/3135	0.94(0.79–1.11)
Quartile4	29/3118	0.75(0.49–1.15)	126/3118	0.79(0.64–0.98)
P for trend		0.34		0.09

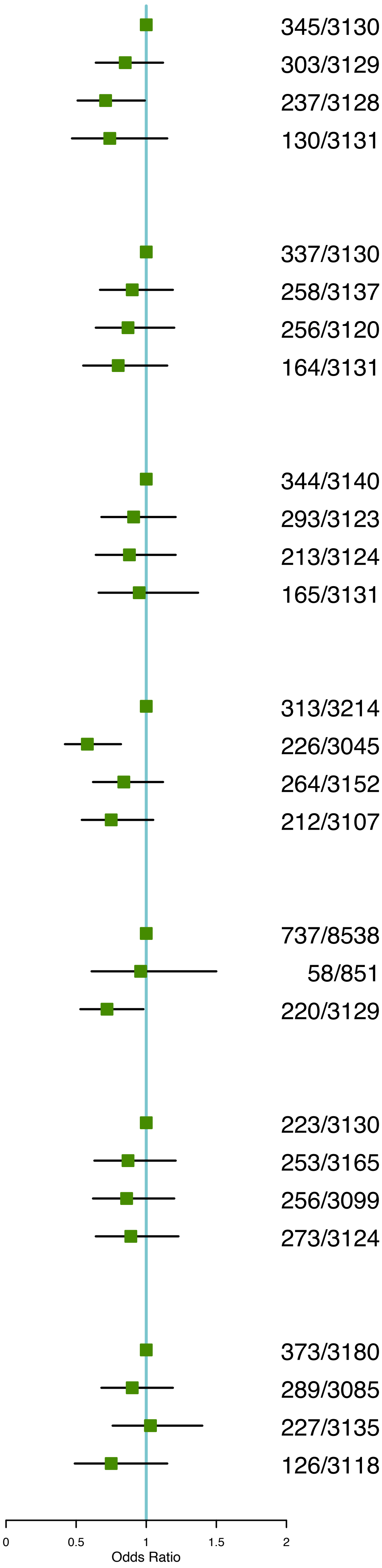

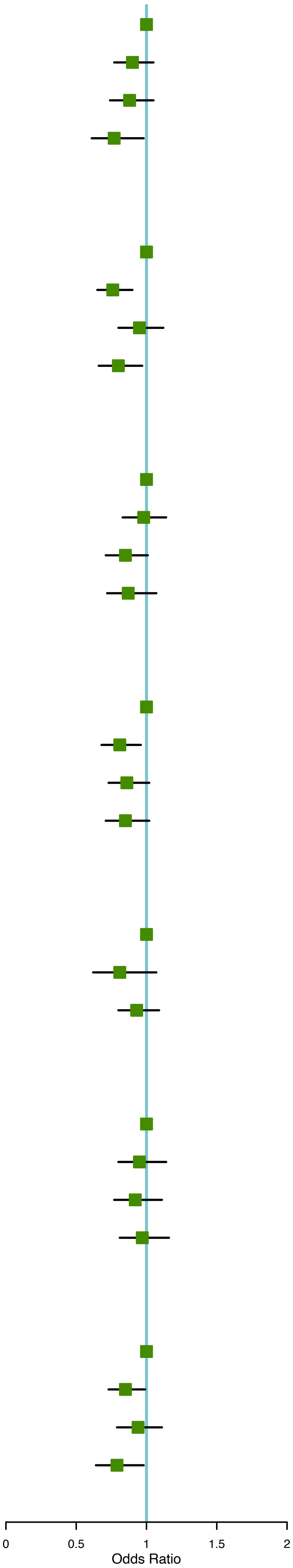

Supplemental Figure 9

Variables	CVD mortality Case/N	CVD mortality HR(95%CI)	ALL-cause mortality Case/N	ALL-cause mortality HR(95%CI)
**Total SPD**				
Quartile1	165/2755	1	501/2755	1
Quartile2	135/2754	0.92(0.73–1.17)	413/2754	0.87(0.76–0.99)
Quartile3	77/2754	0.72(0.54–0.97)	285/2754	0.77(0.65–0.90)
Quartile4	38/2756	0.58(0.38–0.89)	179/2756	0.67(0.54–0.83)
P for trend		<0.01		<0.01
Psex*SPD		0.47		0.13
**Fruit SPD**				
Quartile1	78/2758	1	265/2758	1
Quartile2	114/2753	1.32(0.98–1.78)	381/2753	1.32(1.21–1.56)
Quartile3	119/2758	1.34(1.00–1.80)	369/2758	1.26(1.07–1.48)
Quartile4	104/2750	1.27(0.93–1.74)	363/2750	1.29(1.09–1.52)
P for trend		0.34		0.07
Psex*Fruit SPD		0.27		0.36
**Vegetable SPD**				
Quartile1	135/2767	1	413/2767	1
Quartile2	118/2743	1.01(0.79–1.31)	359/2743	0.99(0.86–1.15)
Quartile3	97/2762	0.93(0.71–1.22)	330/2762	0.96(0.83–1.12)
Quartile4	65/2747	0.74(0.54–1.03)	276/2747	0.91(0.77–1.07)
P for trend		0.07		0.25
Psex*Vegetable SPD		0.95		0.44
**Cereals SPD**				
Quartile1	152/2760	1	463/2760	1
Quartile2	128/2757	0.88(0.69–1.12)	414/2757	0.90(0.78–1.03)
Quartile3	88/2749	0.84(0.63–1.11)	291/2749	0.81(0.69–0.95)
Quartile4	47/2753	0.73(0.50–1.07)	210/2753	0.82(0.68–1.00)
P for trend		0.09		<0.05
Psex*Cereals SPD		0.62		0.59
**Legumes SPD**				
Quartile1	172/2779	1	537/2779	1
Quartile2	130/2732	1.00(0.80–1.28)	426/2732	1.01(0.89–1.15)
Quartile3	86/2752	0.82(0.63–1.07)	271/2752	0.75(0.65–0.88)
Quartile4	27/2756	0.40(0.26–0.62)	144/2756	0.56(0.46–0.68)
P for trend		<0.01		<0.01
Psex*Legumes SPD		0.72		0.19
**Nut SPD**				
Quartile1	250/5820	1	817/5820	1
Quartile2	95/2453	0.91(0.71–1.16)	314/2453	0.87(0.76–1.01)
Quartile3	70/2746	0.71(0.54–0.93)	247/2746	0.71(0.62–0.83)
P for trend		<0.05		<0.01
Psex*Nut SPD		0.28		0.7

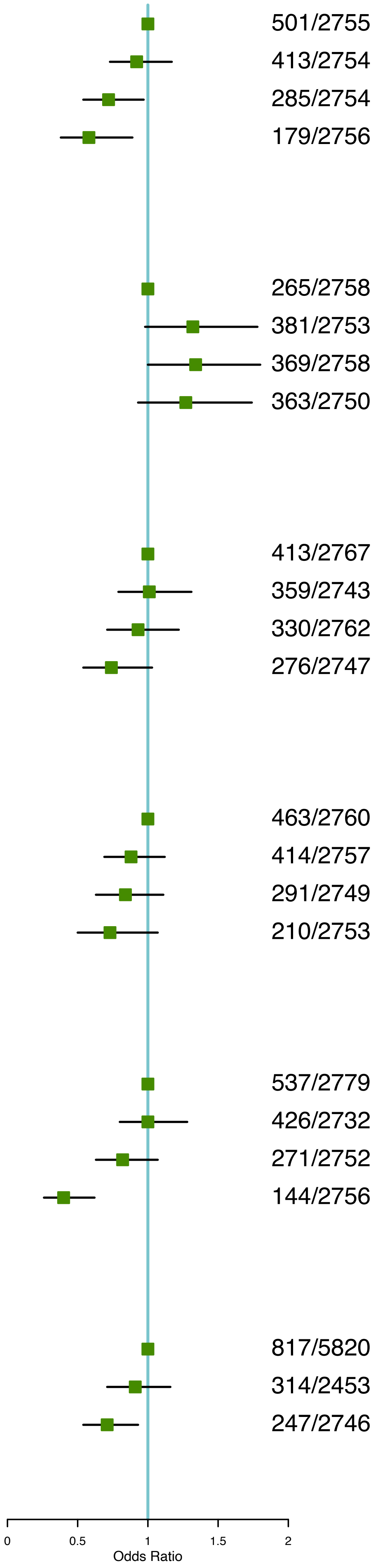

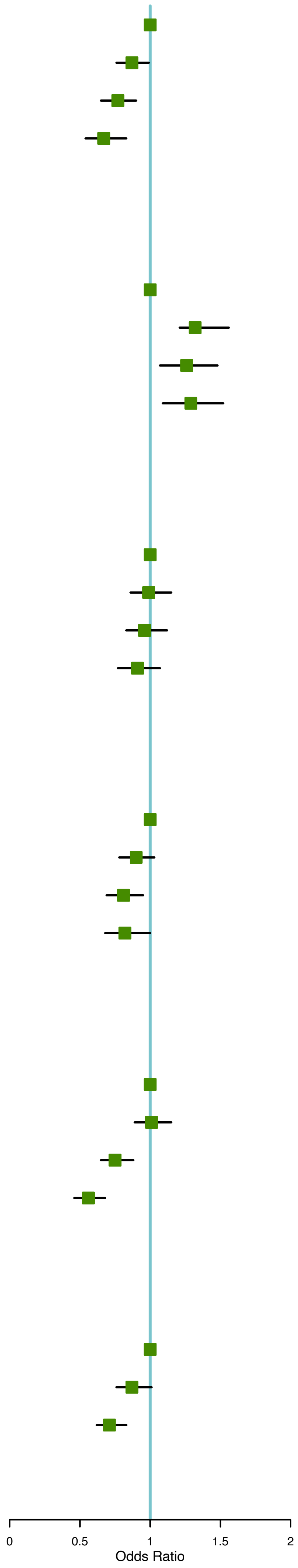

Supplemental Figure 10

Variables	CVD mortality Case/N	CVD mortality HR(95%CI)	ALL-cause mortality Case/N	ALL-cause mortality HR(95%CI)
**Total SPD**				
Quartile1	165/2755	1	501/2755	1
Quartile2	135/2754	0.92(0.73–1.17)	413/2754	0.87(0.76–0.99)
Quartile3	77/2754	0.72(0.54–0.97)	285/2754	0.77(0.65–0.90)
Quartile4	38/2756	0.58(0.38–0.89)	179/2756	0.67(0.54–0.83)
P for trend		<0.01		<0.01
Psex*SPD		0.47		0.13
**Fresh meat SPD**				
Quartile1	136/2769	1	420/2769	1
Quartile2	107/2746	0.86(0.67–1.12)	387/2746	0.98(0.86–1.13)
Quartile3	109/2749	1.16(0.89–1.50)	343/2749	1.07(0.92–1.24)
Quartile4	63/2755	0.99(0.72–1.37)	228/2755	0.93(0.78–1.10)
P for trend		0.63		0.6
Psex*Fresh meat SPD		0.99		0.08
**Cooked meat SPD**				
Quartile1	151/2755	1	464/2755	1
Quartile2	119/2757	0.92(0.72–1.18)	371/2757	0.89(0.78–1.03)
Quartile3	79/2751	0.78(0.58–1.03)	312/2751	0.92(0.79–1.07)
Quartile4	66/2756	0.93(0.67–1.29)	231/2756	0.85(0.71–1.01)
P for trend		0.43		0.09
Psex*Cooked meat SPD		0.3		0.05
**Egg SPD**				
Quartile1	119/2937	1	360/2937	1
Quartile2	86/2572	0.94(0.71–1.25)	314/2572	1.09(0.93–1.27)
Quartile3	107/2787	1.15(0.88–1.50)	343/2787	1.16(1.00–1.35)
Quartile4	103/2723	1.03(0.78–1.36)	361/2723	1.14(0.98–1.33)
P for trend		0.67		0.16
Psex*Egg SPD		0.4		0.57
**Seafood SPD**				
Quartile1	312/7673	1	1012/7673	1
Quartile2	22/593	1.06(0.68–1.64)	76/593	1.06(0.84–1.35)
Quartile3	81/2753	0.86(0.67–1.10)	290/2753	0.90(0.79–1.03)
P for trend		0.22		0.13
Psex*Seafood SPD		<0.01		0.07
**Milk&Yogurt SPD**				
Quartile1	73/2789	1	262/2789	1
Quartile2	93/2789	1.18(0.86–1.61)	320/2789	1.14(0.96–1.34)
Quartile3	112/2686	1.21(0.89–1.64)	364/2686	1.12(0.95–1.32)
Quartile4	137/2755	1.51(1.12–2.03)	432/2755	1.34(1.14–1.58)
P for trend		<0.01		<0.01
Psex*Milk&Yogurt SPD		0.25		<0.05
**Cheese SPD**				
Quartile1	172/2779	1	537/2779	1
Quartile2	130/2732	1.01(0.80–1.27)	426/2732	1.01(0.89–1.15)
Quartile3	86/2752	0.82(0.63–1.07)	271/2752	0.75(0.65–0.88)
Quartile4	27/2756	0.40(0.26–0.62)	144/2756	0.56(0.46–0.68)
P for trend		<0.01		<0.01
Psex*Cheese SPD		0.72		0.19

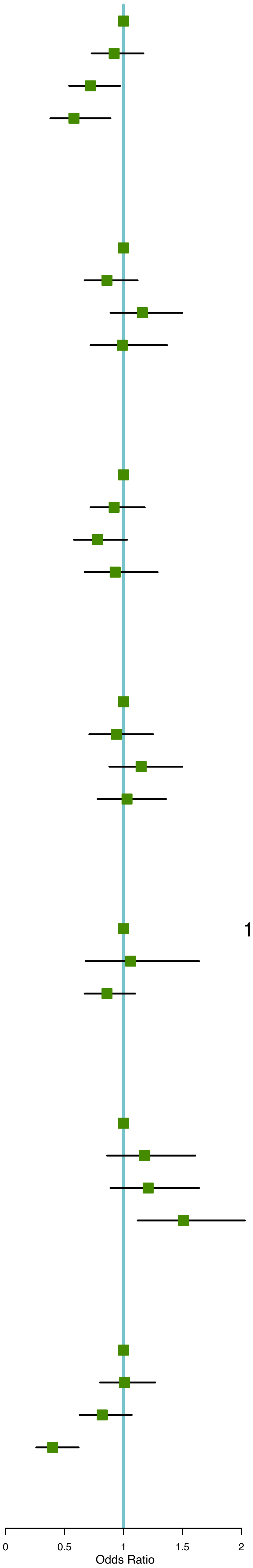

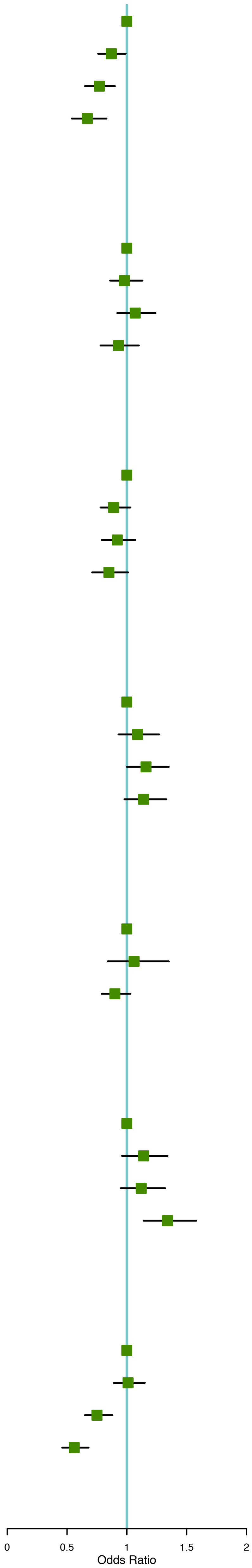

Supplemental Figure 1. Multivariate adjusted hazard ratios (HRs) of the dietary total SPD, fruit-derived SPD, vegetable-derived SPD, cereals-derived SPD, legumes-derived SPD, and nuts-derived SPD with CVD and all-cause mortality in the hypertension subgroup. A logarithmic transformation was performed for non-normal continuous variables. Adjusting factors included age, gender, race, income, education level, regular exercise, smoking, alcohol consumption, BMI(body mass index), total energy intake, AHEI(Alternative Healthy Eating Index), diabetes, and hyperlipidemia.

Supplemental Figure 2. Multivariate adjusted hazard ratios (HRs) of the dietary total SPD, fresh meat-derived SPD, cooked meat-derived SPD, egg-derived SPD, seafood-derived SPD, milk&yogurt-derived SPD, and cheese-derived SPD with CVD and all-cause mortality in the hypertension subgroup. A logarithmic transformation was performed for non-normal continuous variables. Adjusting factors included age, gender, race, income, education level, regular exercise, smoking, alcohol consumption, BMI(body mass index), total energy intake, AHEI(Alternative Healthy Eating Index), diabetes, and hyperlipidemia.

Supplemental Figure 3. Multivariate adjusted hazard ratios (HRs) of the dietary total SPD, fruit-derived SPD, vegetable-derived SPD, cereals-derived SPD, legumes-derived SPD, and nuts-derived SPD with CVD and all-cause mortality in the hyperlipidemia subgroup. A logarithmic transformation was performed for non-normal continuous variables. Adjusting factors included age, gender, race, income, education level, regular exercise, smoking, alcohol consumption, BMI(body mass index), total energy intake, AHEI(Alternative Healthy Eating Index), diabetes, and hypertension.

Supplemental Figure 4. Multivariate adjusted hazard ratios (HRs) of the dietary total SPD, fresh meat-derived SPD, cooked meat-derived SPD, egg-derived SPD, seafood-derived SPD, milk&yogurt-derived SPD, and cheese-derived SPD with CVD and all-cause mortality in the hyperlipidemia subgroup. A logarithmic transformation was performed for non-normal continuous variables. Adjusting factors included age, gender,

race, income, education level, regular exercise, smoking, alcohol consumption, BMI(body mass index), total energy intake, AHEI(Alternative Healthy Eating Index), diabetes, and hypertension.

Supplemental Figure 5. Multivariate adjusted hazard ratios (HRs) of the dietary total SPD, fruit-derived SPD, vegetable-derived SPD, cereals-derived SPD, legumes-derived SPD, and nuts-derived SPD with CVD and all-cause mortality in the male subgroup. A logarithmic transformation was performed for non-normal continuous variables. Adjusting factors included age, race, income, education level, regular exercise, smoking, alcohol consumption, BMI(body mass index), total energy intake, AHEI(Alternative Healthy Eating Index), diabetes, hypertension, and hyperlipidemia.

Supplemental Figure 6. Multivariate adjusted hazard ratios (HRs) of the dietary total SPD, fresh meat-derived SPD, cooked meat-derived SPD, egg-derived SPD, seafood-derived SPD, milk&yogurt-derived SPD, and cheese-derived SPD with CVD and all-cause mortality in the male subgroup. A logarithmic transformation was performed for non-normal continuous variables. Adjusting factors included age, race, income, education level, regular exercise, smoking, alcohol consumption, BMI(body mass index), total energy intake, AHEI(Alternative Healthy Eating Index), diabetes, hypertension, and hyperlipidemia.

Supplemental Figure 7. Multivariate adjusted hazard ratios (HRs) of the dietary total SPD, fruit-derived SPD, vegetable-derived SPD, cereals-derived SPD, legumes-derived SPD, and nuts-derived SPD with CVD and all-cause mortality in the female subgroup. A logarithmic transformation was performed for non-normal continuous variables. Adjusting factors included age, race, income, education level, regular exercise, smoking, alcohol consumption, BMI(body mass index), total energy intake, AHEI(Alternative Healthy Eating Index), diabetes, hypertension, and hyperlipidemia.

Supplemental Figure 8. Multivariate adjusted hazard ratios (HRs) of the dietary total

SPD, fresh meat-derived SPD, cooked meat-derived SPD, egg-derived SPD, seafood-derived SPD, milk&yogurt-derived SPD, and cheese-derived SPD with CVD and all-cause mortality in the female subgroup. A logarithmic transformation was performed for non-normal continuous variables. Adjusting factors included age, race, income, education level, regular exercise, smoking, alcohol consumption, BMI(body mass index), total energy intake, AHEI(Alternative Healthy Eating Index), diabetes, hypertension, and hyperlipidemia.

Supplemental Figure 9. Multivariate adjusted hazard ratios (HRs) of the dietary total SPD, fruit-derived SPD, vegetable-derived SPD, cereals-derived SPD, legumes-derived SPD, and nuts-derived SPD with CVD and all-cause mortality in the <5years subgroup. A logarithmic transformation was performed for non-normal continuous variables. Adjusting factors included age, gender, race, income, education level, regular exercise, smoking, alcohol consumption, BMI(body mass index), total energy intake, AHEI(Alternative Healthy Eating Index), diabetes, hypertension, and hyperlipidemia.

Supplemental Figure 10. Multivariate adjusted hazard ratios (HRs) of the dietary total SPD, fresh meat-derived SPD, cooked meat-derived SPD, egg-derived SPD, seafood-derived SPD, milk&yogurt-derived SPD, and cheese-derived SPD with CVD and all-cause mortality in the <5years subgroup. A logarithmic transformation was performed for non-normal continuous variables. Adjusting factors included age, gender, race, income, education level, regular exercise, smoking, alcohol consumption, BMI(body mass index), total energy intake, AHEI(Alternative Healthy Eating Index), diabetes, hypertension, and hyperlipidemia.
